# Modified Volumetric Modulated Arc Therapy in Left Sided Breast Cancer After Radical Mastectomy With Flattening Filter Free Versus Flattened Beams

**DOI:** 10.1097/MD.0000000000003295

**Published:** 2016-04-08

**Authors:** Youqun Lai, Yanyan Chen, Sangang Wu, Liwan Shi, Lirong Fu, Huiming Ha, Qin Lin

**Affiliations:** From the Department of Radiation Oncology, The First Affiliated Hospital of Xiamen University (YL, SW, LS, LF, HH, QL), and Xiagang Community Health Service Centers, The First Affiliated Hospital of Xiamen University (YC), Xiamen, PR China.

## Abstract

Conventional volumetric modulated arc therapy (C-VMAT) for breast cancer after radical mastectomy had its limitation that resulted in larger volumes of normal tissue receiving low doses. We explored whether there was a way to deal with this disadvantage and determined the potential benefit of flattening filter-free (FFF) beams.

Twenty patients with breast cancer after radical mastectomy were subjected to 3D conformal radiotherapy (3DCRT) and VMAT treatment planning. For VMAT plans, 3 different designs were employed with RapidArc form: conventional-VMAT plan (C-VMAT), modified-VMAT plan (M-VMAT), and modified-VMAT plan using FFF beams (M-VMAT-F). Plan quality and efficiency were assessed for all plans.

For each technique in homogeneity, there were no statistically significant differences. VMAT plans showed superiority compared with 3DCRT in conformity. C-VMAT plans were obviously not only superior to 3DCRT in the medium to high-dose regions (about 15–50 Gy) but also resulted in larger volumes in low-dose regions (about 0–10 Gy). M-VMAT plans were similar to M-VMAT-F. Both of them might significantly reduce the regions of low dose compared with C-VMAT (V5_lung_: ∼ 11.5%; V5_heart_: ∼ 23.8%, *P* < 0.05), even less than 3DCRT in heart irradiation (V2.5_heart_, 9.4%, *P* < 0.05). For liver, contralateral breast, and lung irradiation, M-VMAT-F plans were slightly superior to M-VMAT with a reduction of ∼0.08, 0.2, and 0.24 Gy in the respective mean doses (*P* < 0.05).

C**-**VMAT plans showed superiority compared with 3DCRT, while also resulted in larger volumes of normal tissue receiving low doses. M-VMAT and M-VMAT-F plans might not only reduce the region in the medium to high doses but also have lower volumes in low-dose regions. M-VMAT-F plans were slightly superior compared with M-VMAT due to further contralateral organs sparing.

## INTRODUCTION

Radical mastectomy remains the most-accepted surgical modality in the last decade in many countries,^1^ and radiation therapy is a standard and most important treatment for breast cancer after modified radical mastectomy.^2,3^ Traditionally, 3D conformal radiotherapy (3DCRT) is adopted in a postmastectomy approach with tangential fields for chest wall and separate fields for supraclavicular nodes region. Although tangential beam orientation is optimal for limiting low doses to normal tissues, traditional 3DCRT plans provide inadequate nodal coverage and the conformity of dose distributions is relatively poor.^4^

In recent years, several investigators have studied the role of intensity-modulated radiation therapy (IMRT) for breast cancer after radical mastectomy, and compared dosimetric characteristics of 3DCRT versus IMRT treatment planning techniques.^4–7^ IMRT facilitates to achieve a more homogeneous dose distribution and to decrease normal tissue irradiation by providing more degrees of freedom in the planning process. Nevertheless, the influence of target motion on dose homogeneity and conformity degree will be increased with the increase of beam on times (BOT) for static gantry IMRT.^8,9^ Several authors have investigated the application of volumetric-modulated arc therapy (VMAT) for whole or partial breast treatment.^10–14^ It is clear that VMAT may improve dosimetry and reduce treatment time compared with multiple-field IMRT. However, both IMRT and VMAT will result in increased low doses to large volumes of normal tissue. The effects of an increase in the low-dose region with IMRT or VMAT techniques have to be taken into consideration, for example, whether this will potentially increase estimated risk of secondary cancers. Radiation-induced pulmonary and cardiac toxicity in breast cancer patients have been widely reported by several investigators.^15–17^

In this planning study, we designed a new modified-VMAT plan and evaluated the significance of this technique in left-sided breast cancer after radical mastectomy by comparing with conventional VMAT. With more and more widespread and profound application of TrueBeam^TM^ linear accelerator (TrueBeam SN1402, Varian Medical Systems, Paolo Alto, CA) in radiotherapy, flattening filter-free (FFF) beams have been investigated for breast treatment.^10,18^ We also employed FFF beams for modified-VMAT and determined the potential benefit in breast cancer after radical mastectomy.

## MATERIALS AND METHODS

### Patients and Delineation

The study was approved by the ethics committee of the First Affiliated Hospital of Xiamen University. All patients provided written consent for storage of their medical information in the hospital database and for research use of this information, and the information of patients was anonymized and de-identified before analysis.

Twenty computed tomographic (CT) scans of patients with left-sided breast cancer involving supraclavicular nodes, who underwent radical mastectomy, were selected for this treatment planning study. All patients underwent a planning CT scan with 5 mm slice thickness (General Electric Medical Systems (GE Healthcare, USA), CT Lightspeed 16).

**FIGURE 1:**
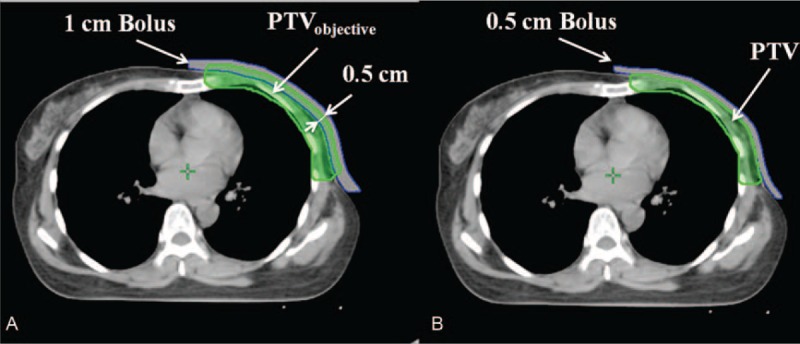
Delineated planning target volume in breast cancer of radical mastectomy for optimization. (A) A 1-cm bolus was inserted and the PTV_objective_ was as the objective structure during optimization. The PTV_objective_ was derived from PTV along with a 5-mm margin on the skin surface around PTV. (B) For final dose calculation and analysis, the 1-cm bolus was replaced with a 0.5-cm bolus.

Clinical target volume (CTV) was defined by the entire ipsilateral chest wall along with supraclavicular nodes region. The planning target volume (PTV) was added a 5-mm margin around the CTV. Mean PTV size and standard deviation were 612.6 ± 138.7 cm^3^ (range: 443.9–825.8 cm^3^). The PTV_objective_ was derived from PTV along with a 5-mm margin on the skin surface around PTV (Figure 1A). Organs at risk (OAR), such as ipsilateral lung, heart, contralateral breast and lung, and liver, were outlined in the axial CT sections. For optimization and analysis purposes, a 1-cm bolus was applied to the skin surface around PTV to prevent the optimizer compensating for lack of dose in the buildup region during optimization. Considering the influence of physiological motion, the PTV_objective_ was as the objective structure during optimization to reduce uncertainties in dose delivery. Before the final dose calculation,^18^ the 1-cm bolus was replaced with a 0.5-cm bolus (Figure 1B).

### Treatment Planning

Treatment plans were generated for a TrueBeam linac, equipped with standard Millennium MLC with 120 leaves (0.5 cm spatial resolution at isocenter in the inner 20 and 1.0 cm spatial resolution for the 2 × 10 cm outer length of the field). Four techniques (3DCRT, C-VMAT, M-VMAT, and M-VMAT-F) for treatment plans were designed for all patients as described below. For VMAT techniques, treatment planning was performed in the Eclipse treatment planning system (Varian Medical Systems, Paolo Alto, CA, PRO 11.0, AAA 11.0) using 6X-FF or 6X-FFF beams. The maximum dose rate of 600 MU/min for 6X-FF beams and 1400 MU/min for 6X-FFF beams was selected. The prescribed dose (PD) was 25 × 2 Gy (50 Gy) and plans were normalized so that 95% of PTV received 95% of the PD. The same objectives were used for each RapidArc plan, and to minimize the volume inside the PTV receiving >107% of the dose. The Normal Tissue Objective automatic tool in Eclipse TPS was used to minimize dose spread outside the PTV. For the OARs, the mean dose for ipsilateral lung was received <15 Gy and V_20__Gy_ < 22%.

### 3D Conformal Technique (3DCRT)

3DCRT plans were designed with 4 fields, using 6MV photon beams, with 2 wedged tangential fields for chest wall and 2 wedged separate fields for supraclavicular nodes region. Each field included 0 to 2 subsegments shaped by multileaf collimators (MLCs) to ensure the D_max_ of PTV not more than 107% of the PD.

### Conventional VMAT Plans With RapidArc form (C-VMAT)

**FIGURE 2:**
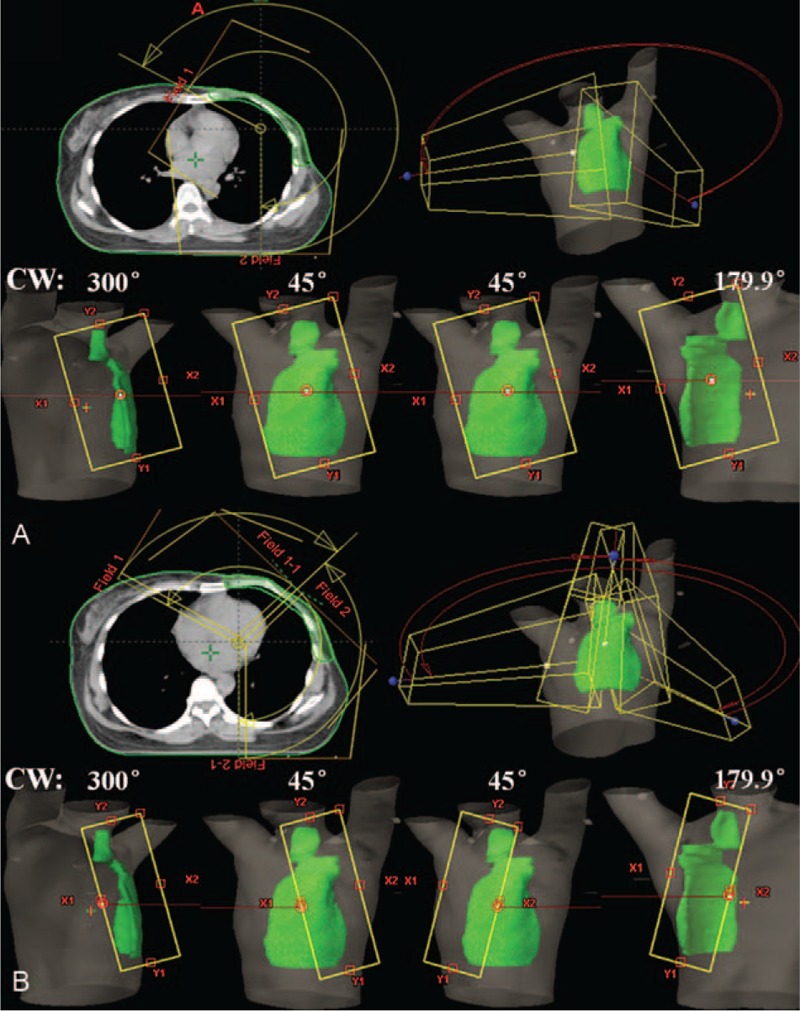
(A) Beam setup of conventional-VMAT (C-VMAT) plans: double ipsilateral partial arcs with a maximum individual length of 240°. (B) Beam setup of modified-VMAT (M-VMAT) plans: each 240° arc was divided into 2 sections.

For C-VMAT plans,^8^ as shown in Figure 2A, double ipsilateral partial arcs with a maximum individual length of 240° starting from the mid-stermum were adopted in this study. In clockwise direction (CW), collimator angles were ranged from 15° to 30°, and 6MV photon beams were used. Similarly, in counter-clockwise (CCW) direction, the collimator settings were kept constant.

### Modified VMAT Plans With Half-field Technique (M-VMAT)

Figure 2B shows the beam setup of modified VMAT plans. One 240° arc was divided into 2 equal sections covering 120° each. In CW rotation, the collimator angle was set to 15° to 30° and a half-field was opened at X2 of the collimator for the first part of arc, while in second part of arc, the collimator angle was 345° to 330° and a half-field was opened at X1 of the collimator. In CCW rotation, the collimator settings were kept constant. For M-VMAT-F plans, 6X-FFF beams were used and the same beam settings were applied. The maximum dose rate was set to 1400 MU/min.

### Plan Evaluation and Statistical Tools

For the quantitative evaluation of the plans, the standard dose volume histograms (DVHs) were used. The values of D_98%_ and D_2%_ (dose received by 98% and 2% of the PTV) for the PTV were defined as metrics for minimum and maximum doses. The conformity index (CI) was defined as: CI = (V_PTV_/TV_PV_)/(TV_PV_/V_TV_). V_PTV_ is the volume of PTV. TV_PV_ is the portion of the V_PTV_ within the 95% of prescribed isodose line. V_TV_ is the volume of the body that received 95% of the PD. The homogeneity index (HI) was defined as: HI = D_5%_/D_95%_ (dose received by 5% and 95% of the PTV).^19^ For OARs, the mean doses, and a set of appropriate V_x(Gy)_ and D_y(%)_ values to ipsilateral lung, heart, contralateral breast and lung, and liver were analyzed. To evaluate the efficiency of each technique, total MUs, BOT, and mean dose rate [monitor unit (MU)/min)] were compared.

Statistical analyses were performed in order to compare the different techniques using a paired *t* test. *P* value ≤0.05 was considered statistically significant.

## RESULTS

### PTV Coverage and Dose Distribution

**FIGURE 3:**
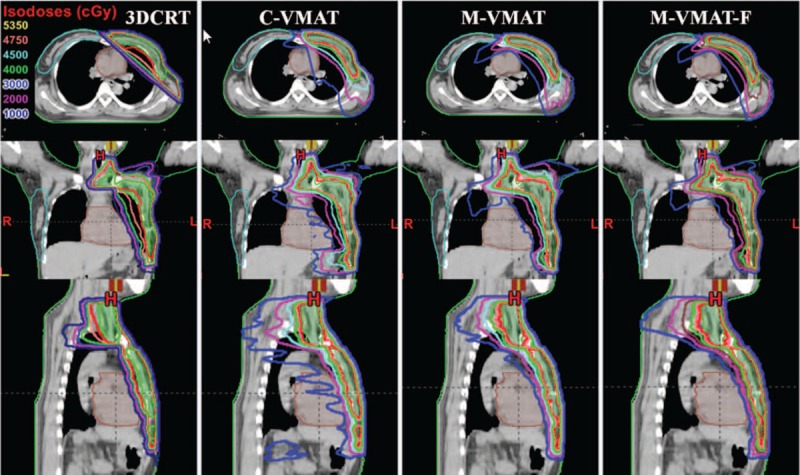
Isodose distributions for 1 patient with breast cancer after radical mastectomy in axial, coronal, and sagittal planes. 3DCRT = 3D conformal technique, C-VMAT = VMAT plans with RapidArc form in Eclipse treatment planning system, M-VMAT = modified-VMAT plans with RapidArc form, M-VMAT-F = modified-VMAT plans using flattening filter-free (FFF) beams.

Table 1 presented dosimetric parameters of PTV for all 4 groups of treatment plans created with different planning techniques. No substantial differences were observed between the 4 treatment plans in homogeneity, while VMAT plans showed superiority compared with 3DCRT in the conformity. Transversal, coronal, and sagittal dose distributions are displayed in Figure 3 for 1 patient with left-sided breast cancer after radical mastectomy. It was evident that C-VMAT plans would result in larger volumes of normal tissue receiving low doses compared with 3DCRT plans. Dose distributions in M-VMAT plans were much better than C-VMAT, and M-VMAT-F plans were similar to M-VMAT. Both M-VMAT and M-VMAT-F plans might not only reduce the region in the medium to high doses but also have lower volumes in low-dose regions for normal tissue.

**TABLE 1:**
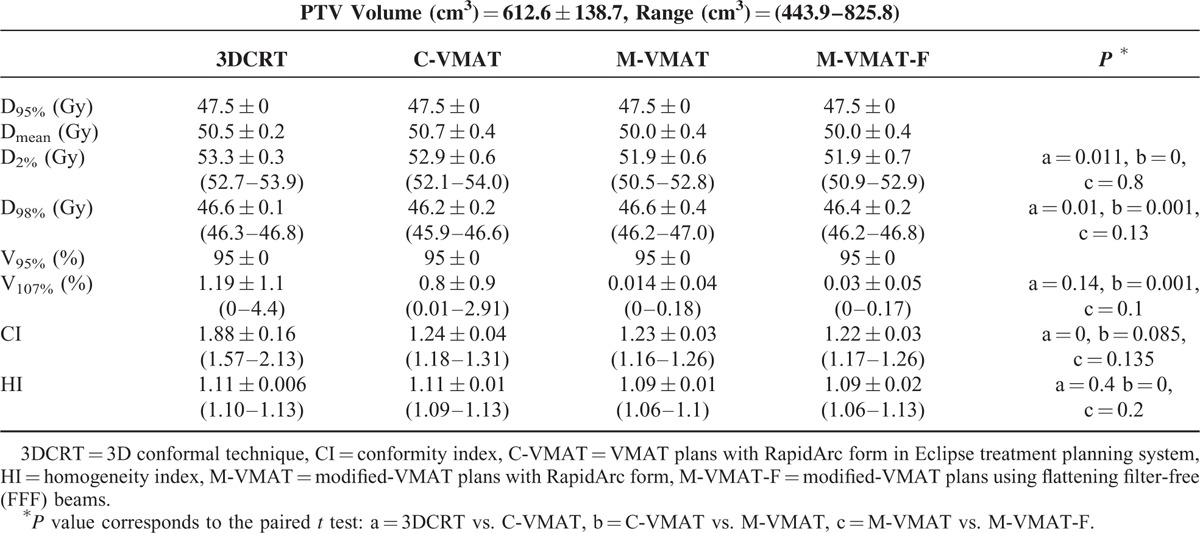
Dosimetric Parameters of PTV for Treatment Plans Created With Different Planning Techniques

### Dose to Organs at Risk

**FIGURE 4:**
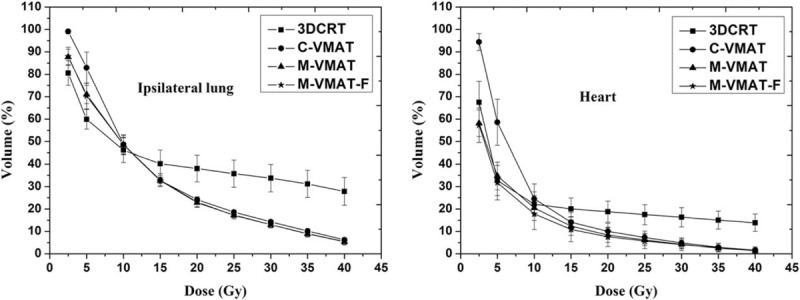
Average dose-volume histogram (DVH) comparison for ipsilateral lung and heart, with different planning techniques. 3DCRT = 3D conformal technique, C-VMAT = VMAT plans with RapidArc form in Eclipse treatment planning system, M-VMAT = modified-VMAT plans with RapidArc form, M-VMAT-F = modified-VMAT plans using flattening filter-free (FFF) beams.

Figure 4 shows average dose-volume histogram (DVH) comparison for ipsilateral lung and heart with different planning techniques. For ipsilateral lung and heart irradiation, C-VMAT plans were obviously not only superior to 3DCRT in the medium to high-dose regions (about 15–50 Gy) but also resulted in larger volumes in low-dose regions (about 0–10 Gy). M-VMAT plans were similar to M-VMAT-F, and both might significantly reduce the regions of low dose compared with C-VMAT (V5_lung_: ∼11.5%; V5_heart_: ∼23.8%, *P* < 0.05), even less than 3DCRT in heart irradiation (V2.5_heart_, 9.4%, *P* < 0.05). That is, for heart irradiation, M-VMAT and M-VMAT-F plans might not only reduce the region in the medium to high doses but also have lower volumes in low-dose regions than 3DCRT.

Table 2 presents the results of DVH numerical analysis for the organs at risk: ipsilateral lung, heart, contralateral breast, contralateral lung, and liver. For the irradiation of liver, contralateral breast and lung, M-VMAT-F plans were slightly superior to M-VMAT with a reduction of ∼0.08, 0.2, and 0.24 Gy in the respective mean doses (*P* < 0.05). However, in M-VMAT plans, the V5_lung_ and V20_lung_ were increased by ∼4% and 0.7%, respectively, compared with C-VMAT plans for contralateral lung irradiation.

**TABLE 2:**
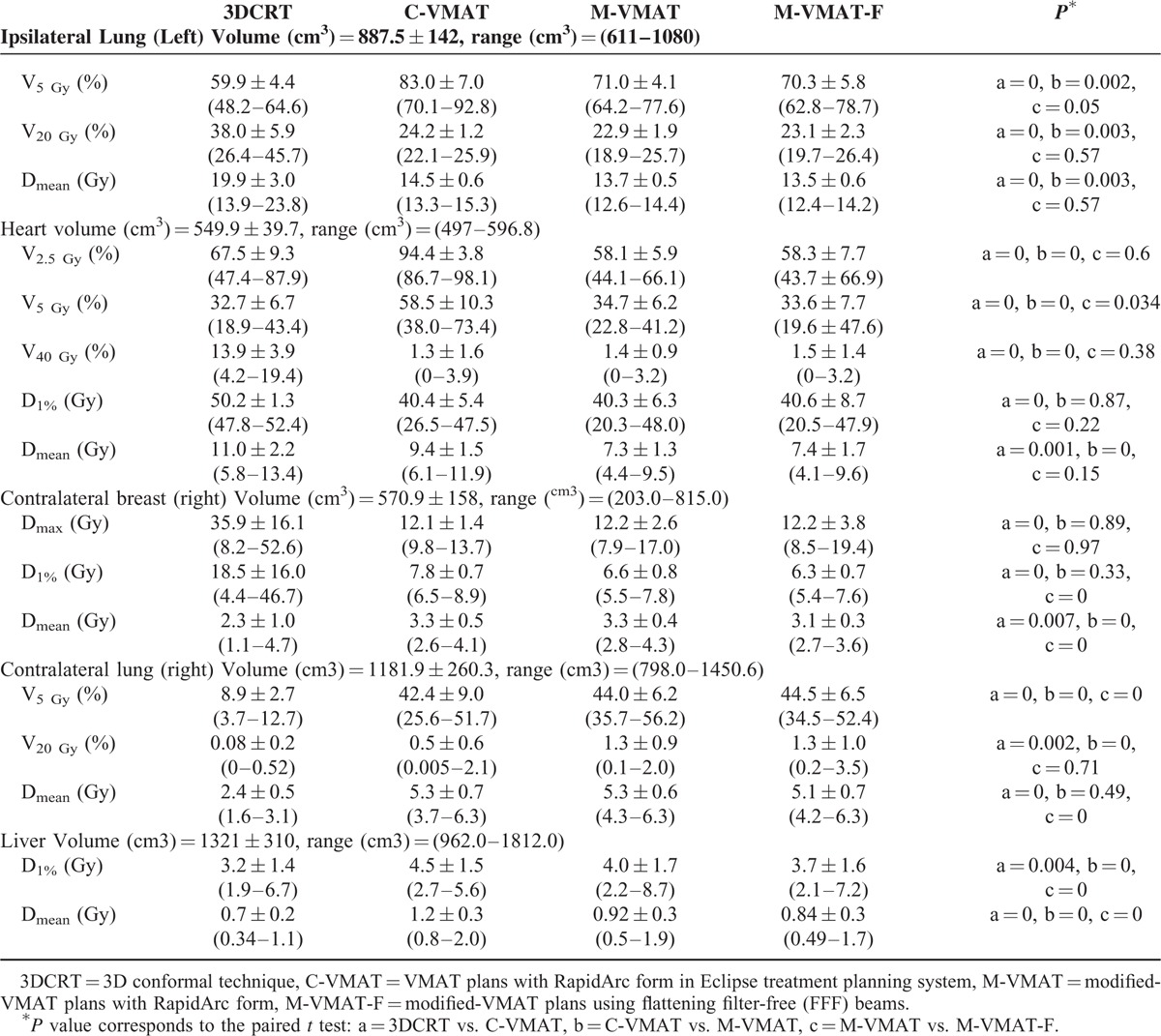
Results of Dose-Volume Histogram (DVH) Numerical Analysis for the Organs at Risk: Ipsilateral Lung, Heart, Contralateral Breast, Contralateral Lung, and Liver

### MU and Beam Delivery Time

Table 3 summarizes the results for all treatment plans about the number of monitor units (MU), BOT, and mean dose rate (MDR). Beam delivery times were similar for each technique. The total MUs for M-VMAT plans were increased by an average of 7.2% compared with C-VMAT. For M-VMAT-F plans, the mean MUs was 839 MUs, representing an average of 25% increase compared with M-VMAT.

**TABLE 3:**
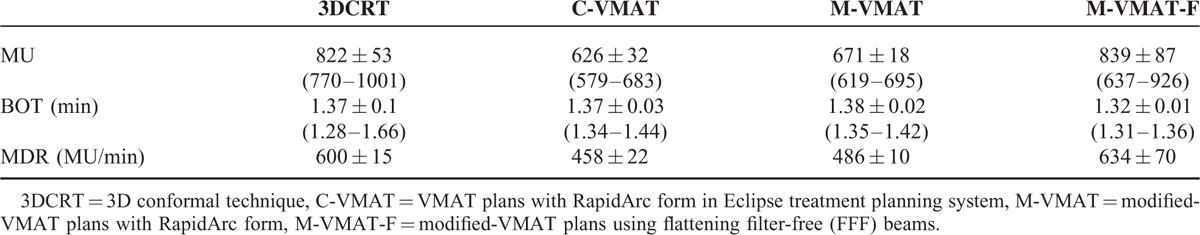
The Number of Monitor Units (MU), Beam-on Time (BOT), and Mean dose Rate (MDR) for Treatment Plans Created With Different Planning Techniques

## DISCUSSION

The present study addressed a comparative appraisal of 4 different techniques using flattened or FFF beams for left-sided breast cancer after radical mastectomy. For radiotherapy of chest wall and supraclavicular nodes region, traditional 3DCRT is still a common treatment technique in many countries. However, due to inadequate nodal coverage and poor conformity of dose distributions (Table 1), the radiation-induced skin injury was likely to be observed in many patients, especially injury of armpit skin. As expected with a rotational technique, C-VMAT plans resulted in larger volumes of normal tissue receiving low doses compared with 3DCRT (Figure 3). This was no difference compared with earlier investigations.^8^

In our study, a new modified-VMAT plan (M-VMAT) using flattened or FFF beams was designed, and the plan quality and efficiency were assessed for left-sided breast cancer after radical mastectomy. For more reasonable dosimetric comparison of different techniques, the same objectives were used for each VMAT plan. The data summarized in this report demonstrated that the dose distributions in M-VMAT plans were better than C-VMAT as clearly visible in Figure 3. As shown in Figure 4, the M-VMAT plans could provide superior ipsilateral tissue (ipsilateral lung and heart) sparing, for that it might not only reduced the region in the medium to high doses but also had lower volumes in low-dose regions.

The more advantage to M-VMAT plans than C-VMAT using same energy beams (6X-FF beams) can be attributed to the differences in the beam setup. The most important difference between these 2 VMAT plans was that half-field beam technique was employed for M-VMAT plans during the whole process of target volume irradiation. It is well known that an independent jaw can be moved to block off half of the field along the central axis to eliminate beam divergence. This feature is useful for adjacent normal healthy tissue sparing, that is, ipsilateral lung and heart sparing in radiotherapy for left-sided breast cancer. To achieve goal of half-field beam during the whole process of irradiation, the design of M-VMAT plan is shown in Figure 2B. That is, in CW rotation, a half-field was opened at X2 of the collimator for the first part of arc from 300° to 60°, while in the second part of arc, a half-field was opened at X1 of the collimator from 60° to 179.9°. Therefore, the difference between 2 VMAT plans was that collimator angle and field were changed in second part of arc in M-VMAT plan. By this way, half-field beams were always used during the whole process of target volume irradiation. However, a possible drawback for this approach was the fact that the V5_lung_ and V20_lung_ were increased for contralateral lung irradiation compared with C-VMAT plans (Table 2). The effects of an increase in contralateral lung V_5_ and V_20_ (∼4%, 0.7%) have to be weighed against the advantage of reduction in superior ipsilateral lung and heart sparing.

FFF beams show their unique characteristics for a higher dose rate and lower peripheral dose. We evaluated the dosimetric benefits of FFF beams compared with flattened beams. Figure 3 shows that the dose distributions in M-VMAT-F plans were similar to M-VMAT. However, for the irradiation of liver, contralateral breast, and lung, M-VMAT-F plans were slightly superior to M-VMAT with a reduction of ∼0.08, 0.2, and 0.24 Gy in the respective mean doses (*P* < 0.05). Whether this feature makes any sense for the treatment of left-sided breast cancer after radical mastectomy using FFF beams is still worth exploring. In addition, we observed that the average dose rate in M-VMAT-F plans was only 634 MU/min (Table 3), though the maximum dose rate could reach 1400 MU/min. About the beam delivery times, the BOT was similar for each VMAT technique, as the VMAT BOT was limited by the gantry speed. Because of this, the potential for a higher dose rate of FFF beams could not be exploited in conventional radiotherapy (2 Gy/fraction).

## CONCLUSIONS

The results demonstrate that with respect to the radiotherapy of chest wall and supraclavicular nodes region for left-sided breast cancer after radical mastectomy, C**-**VMAT plans not only showed superiority compared with 3DCRT while also resulted in larger volumes of normal tissue receiving low doses. Dose distributions in M-VMAT plans were much better than C-VMAT, and M-VMAT-F plans were similar to M-VMAT. For ipsilateral lung and heart irradiation, both M-VMAT and M-VMAT-F plans might not only reduce the region in the medium to high doses but also have lower volumes in low-dose regions. By use of FFF beams, M-VMAT-F plans were slightly superior compared with M-VMAT due to further contralateral organs sparing.
